# Things Old, New, and Useful in the Operating Room

**Published:** 1893-07

**Authors:** A. C. Hewitt


### THE DENTAL REGISTER.

VOL. XLVII.] JULY, 1893. [No. 7.

### COMMUNICATIONS.

### Things Old, New and Useful in the Operating Room.

BY A. C. HEWITT., D.D.S.

Read before the Joint Meeting of the Illinois and Iowa State Dental Societies, June 10th, 1893.

(ABSTRACT.)

"Things old, new and useful in the operating room," broad enough to inspire a book, has not led me to that attempt. In the little I have to say I have avoided the topics named in the program assigned to others.

Begging, therefore, the indulgence of the sages of the Societies I will address myself chiefly to the younger members of the Societies, saying something about Detergents, Antiseptics, and Obtundents. Not at all in a scientific way, but formulating some materials,–"things" that I have found useful in every day office practice.

Clean, soft hands, whether old or new, are still useful. The successful busy dentist must sometimes handle flash, file, and forge; he must occasionally hold the crucible, the die and the scraper; he must sometimes grind at the lathe, and polish at the buffer. His hands become soiled. Soaps? Yes, if scentless; or at most, of the daintiest odor. These will not always whiten. The following is excellent:

| | | |
|---|---|---|
| ℞ | Pulv. Acidum Boracicum, - - | 1 lb. |
| | " Sodae Carb. - - | 2 lbs. |
| | " Pumace - - - | 1 lb |

Glycerine Qs to form paste.

Sig. Use as a soap.

If hands are stained, as with nitrate of silver, wet the stains and rub with Cyinide of Potassium, then wash. Never apply soap first, as that sets the stain. This paste is searching in action. An emollient should follow, and as perfumes are not always agreeable to the patient, especially in the mouth, nothing is better to soften, smooth, and render the hands agreeable than the following lotion:

℞ Fowlers Solution,

Liq. Potassae Arsenitas, Fl. ℥ iv.

Glycerine, - - - Fl. ℥ iij.

Bay Rum, - - Fl. ℥ xvj.

Aq. Pura, ad Fl. ℥ xxxij.

Sig. Lotion for hands, face, etc.

After washing with the paste and drying with a towel, moisten the hands with the lotion and let it evaporate. In making this lotion use only the best of Bay Rum; that distilled from the bay leaf, not bay oil and alcohol.

ANTISEPTIC SPRAY.

Operators run great risks from working over patients suffering from La Grippe, Catarrh, (nasal) incipient Diphtheria, Scarlatina, not to mention syphilitic taints and Tuberculosis.

If a hint of any of these is present, before operating some precaution should be taken. A spray prepared to be used from the atomizers of the drug stores is of special value, prepared as follows:

℞ Antiseptic Pastilles (Carl Seilers formula Pastilles.) ss.
Aq. Pura, - - - Fl ℥ j.
Sol. Hyd. Chlor. Cocaine 4 per cent, F ℥ ss.
Glycerine, - - - - - Fl ℥ ij.
Sig. Use as a spray.

Crush and dissolve the pastille first in water—boiling water is quicker, then add cocaine. It will turn milky and cloudy. Add glycerine, which will immediately make the liquid clear. Spray nose, mouth and throat, and you will be safe. This spray is admirable to benumb sensitive gums, before using the gilling-twine to hold rubber dam in place. It is exceedingly useful also in allaying irritability of soft palate in taking impressions.

A convenient way to make and keep a 4 per cent solution of cocaine is

| | | | |
|---|---|---|---|
| ℞ | Hyd. Chlor. Cocaine, | - | grs. xxxviij. |
| | Glycerine, - - | - | Fl. ℥ ij. |
| | Aq. Pura, | ad | Fl. ℥ ij. |
| | M. | | |

Thus prepared and covered from the light the solution will keep an indefinite time.

An admirable, safe, efficient cleanser of instruments, antiseptically, is hydronapthol. I am indebted to Prof. Harlan for the suggestion that has led to the use of this valuable drug.

| | | | |
|---|---|---|---|
| ℞ | Alcohol, | - - - | Fl. ℥ ij. |
| | Hydronapthol, | - - - | grs. xx. |
| | M. | | |

Put into a wide mouthed bottle, as a quinine bottle. Dip your instrument, whether excavator, forceps, searcher, or pyorrhea blade into the liquid, and lay away to dry. The most delicate steel will not be tarnished by it and it needs no wiping.

Hydronapthol as a dressing to septic root canals has few equals in value. I think there is nothing superior or more convenient:

| | | | |
|---|---|---|---|
| ℞ | Olei Caryophylli. | | |
| | Olei Cassia (Buds) aa, | - - | Fl. j. |
| | Hydronapthol, | - - | grs. xx. |
| | M. | | |

Sig. Antiseptic for root canals.

Again thanks to Prof. Harlan for the suggestion leading to the formula.

Eucalyptol (Sanders & Sons Eucalyptol e fl,—do not mistake oil of Eucalyptus wood for the foregoing.)

If you wound your hands and fear blood poisoning at once apply the Eucalyptol, and cover with chlora-percha, and you will be safe. If for any reason you wish to extract a tooth and re-plant (to trim a node causing neuralgia, or to remove from a foramen a broken broach,) after extraction, holding the tooth in the forceps dip it in Eucalyptol, trim and smooth the root, enlarge the root canal through the foramen, fill the canal, re-dip the tooth in Eucalyptol, put it back into its socket, driving it well home, and you will tire of waiting for suppuration or root absorption. On no account touch the tooth with water, and if possible keep the socket free from saliva. Re-plant through the fibrinated blood that lies in the socket. In my surgical practice I have learned not to apply water to any wound where demanded for removing dirt. Blood and serum, kept aseptic, are nature's provision for healing by first intention.

OBTUNDENTS.

There is so strong a desire on the part of the patients for "painless dentistry" and such a stress on the part of the operator to avoid giving pain, that I think I may be pardoned if I occupy some of your time upon this subject. Quacks advertise obtunders both for sale and for use. I have taken some pains to investigate some of these compounds, they being furnished by sample. That some of them possess the property of local anæsthetics cannot be denied. And I have found that just in proportion to the effectiveness of the drugs the mixtures contain more or less cocaine in one form or another, even though advertised not to be made from any of the derivatives of the cocoa plant. So strikingly has this been the result of my experiments that I have been led to compound an obtundent. The result has been most gratifying. My chief reliance has been on the hydrochlorate of cocaine. Before giving you the formula that I employ I will detail a case or two by permission of my patients. Miss May Cleo Smith, D.D.S., of Kenosha, Wis., had a left superior bicuspid root that she wished removed. Gas? "No." Ether or Chloroform? Emphatically "No." I suggested my obtundent. "Yes, she would like that if I thought best." Her willingness and the growing pallor of the mouth and lips induced me to try it. I applied it in a way I shall describe, and removed the root, by no means an easy operation. Process and tissues were as firm as her will. I will describe the operation that you may be the better judge: Using a sharp-bladed forceps I thrust the outer blade along the root under the gum over the un-absorbed process, cutting my way to the point I judged sufficient, I passed the inner beak over gum and border that I might get a sufficient hold for traction. After the root was removed she expressed the utmost gratification.

She said, "It did not hurt though I expected it would." "I want your formula if you will favor me." Not a nervous person (hysterically,) but of a fine organization, capable of suffering, clear headed, conservative in her character, her words are weighty.

Mrs. P. had several teeth extracted, preparatory to a full upper denture. Averse to anæsthetics, she yet readily assented to the Obtundent. I think seven teeth were to be removed. "Between times" she unaided leaned over the cuspidor and freed her mouth of blood. After the teeth were out she expressed the utmost satisfaction. To repeat the substance of her words, "That is better than gas." "I could feel the forceps grasp the teeth but it did not hurt. I kept expecting it would hurt, but it was painless." In this case I gave from the mouth of this bottle, about four inhalations of chloroform. She was not aware she was taking the anæsthetic. By the same method as in the last case I extracted for another lady three solid roots, one a superior cuspid, and a cuspid tooth. The latter broke at the border, and I had to "reach" for the root. After the operation the patient, a large strong woman, said "Oh that is delightful! I am going to tell Mr.——who has teeth to extract, no need of gas." I might add case after case to the same purport, but the above are sufficient.

The relief from shock, the help to operator and patient, has led me to its permanent adoption. I give you the formula and my mode of application. Perhaps the eminent in our profession may suggest an improved formula. I certainly should receive suggestions with much gratitude. I have been not a little perplexed to find a name that is at once suggestive, explanatory and honest. For want of a better and for the present I will name it *Compound Cocaine Pigment.*

| | | |
|---|---|---|
| ℞ | Cocaine Hydrochlorate, - | gr. cxx. |
| | Atropinæ, - - - | gr. $\frac{1}{10}$. |
| | Stropanthin, - - - | gr. $\frac{1}{5}$. |
| | Hydronapthol. - - | gr. x. |
| | Olei Caryophylli, - - - | F. ij ʒ. |
| | Olei Cajputi, - - - | F. ij ʒ. |
| | Glycerine, ad | F. j ʒ. |
| | M. | |

Sig. Use with applicator as local anæsthetic.

The cocaine should be in characteristic prisms, of the smaller rhombic system, and large clean crystals; as that sold in fine powder is often adulterated with morphine and other substances to give it weight. Pulverize the cocaine before mixing to hasten solution.

In Prof. Ingall's work "Diseases of the Chest, Throat and Nasal Passages," is to be found a formula for local anæsthesia, which is as follows and which I give by his permission:

| | | |
|---|---|---|
| ℞ Atropinæ | - - - | gr. $\frac{1}{10}$. |
| Stropanthine, | - | gr. $\frac{1}{5}$. |
| Cocaine Hyd. Chlor, | - - | gr. xx. |
| Acid Carbolic, | - - | gr. x. |
| Olei Caryophylli, | - - - | M. iij. |
| Aq. Dist. | ad | Fl. ℥ j. |
| M. | | |

Sig. Local Anæsthetic.

Dr. Ingalls informed me that the formula had been used under copyright, advertised for "painless dentistry etc." The right to use sold at large figures, etc., etc. He also said that he deemed it safe as a "hypodermic" to the extent of five minims, and that its local anæsthetic effect was marked and reliable.

A good way to apply the "Pigment" is, first to bathe the gums surrounding the teeth to be extracted, with alcohol and hydronapthol to remove mucous and all foreign substances from the gums and membrane surrounding the tooth, neck and roots. This is important. Second with warmed wax or modelling compound take an impression of the teeth or roots. Remove from the mouth and slightly enlarge and deepen the impression of the several teeth or roots to be drawn. Place a small pledget of absorbent cotton, dipped in the Pigment, in the enlarged tooth and root impressions, and carry to the mouth, which, after the alcohol, should be made as dry as possible. Press firmly home and hold there from four to ten minutes. Three things are essential to success: clean gums and necks of teeth, absence of saliva, and close contact by the mixture. Do some of you say "That it is too much fuss, too much nicety of preparation?" To such I reply, Do not use it, you will fail. No one will succeed in any operation in the largest sense without minute care at every step. The minuteness, the care, or their lack, mark the difference between the skilled operator and the bungler.

This brings me naturally to the consideration of a "Thing" and "Object of thought" more sinned against, more abused, a substance in the hands of the unskilled and the reckless, as dangerous to human life as Prussic Acid, or Dynamite, but used properly, legitimately, as safe as the odor from the heart of a rose. Neither "New" nor "Old" strictly, in a general way known to everybody, but scientifically as little mastered both in substance and action as the nebulæ of the Milky Way, Chloroform the king of Obtundents. I use the word Obtundent rather than Anæsthetic in speaking of chloroform in connection, because it seems to limit its use to a degree less than is commonly attached to chloroform. Please remember that I am not using terms with exact meanings.

I shall not abuse the privilege of this Convention by attempting a learned discussion of its discovery, manufacture, purification, chemical components, nor of its mode of action as an *anæsthetic*, its toxic action, nor of the modes of restoration to be used when an over-dose is given. With all these you are doubtless familiar.

The more clearly to define my attitude in relation to chloroform as an anæsthetic, I wish to say that there is not in all the range of operative dentistry, and in the demand of oral surgery, more than four to six operations demanding or justifying its exhibition, to complete anæsthesia. The obtundent influence is ample. So well convinced am I of this, that I do not hesitate to say that in case of death under the hands of any dentist (not skilled as an Oral Surgeon and supported by skillful counsel) Indictment, Conviction and Imprisonment would surely follow if a thorough intelligent prosecution were pressed. Under no circumstance is a dentist justified in fully anæsthetising a patient for extraction of teeth or for minor operations of oral surgery. An incident occurred to the writer of this paper while he was a medical student, aged about twenty-two years, which made a profound impression. A right inferior molar became painful beyond endurance. Under the obtundent influence of chloroform he "lanced the gums" as was then taught, and extracted his own tooth without pain. Since that time he has availed himself of its beneficence with growing wonder at each recurring miracle. His faith has grown until it has become a verity. During a somewhat lengthened practice never an accident, or an approach to one, has occurred, so far as he knows, though used many thousand times. As a result of careful study and extensive use he does not hesitate to commend its general use always as an *Obtundent.* (Please observe the emphasis of that word.) When given as I shall describe, it is safe for the young and aged, the robust and feeble, the sick and the healthy, the nervous and the stolid. Even the insane yield to its charm with a delight that is pathetic to witness. Thus used as a temporary alleviator of pain, however intense, chloroform has no known rival.

HOW ADMINISTERED.

Having tested almost numberless devices, from a sponge to an elaborate machine, I have chosen a means so simple as to be almost ludicrous. A wide mouthed half ounce to ounce bottle. An ordinary morphine bottle is as good as any. Any glass bottle two and one half inches high, an inch and one half diameter, with mouth three fourths of an inch across will do. Of course, it should be *clean.* If the chloroform is to be kept in the bottle after administration the cork or stopper should be hermetical, and the bottle wrapped in dark paper, and kept in a dark place. The chloroform should be pure, never of a doubtful manufacture. No preparation of the patient is necessary, except that an empty stomach is to be preferred. Or if the drug is to be given soon after a meal the food should be light in quality and quantity; otherwise if the *Obtundent* effect is pushed to, or near the anæsthetic line slight nausea may supervene, the only ill effect I have observed even with the stomach overloaded.

Place not to exceed a teaspoonful of chloroform in the bottle. With it open, place it near one nostril of the patient, nearly on a level with the nose, remembering that the vapor of chloroform is heavier than the atmosphere, and the narcotized air tends to fall. Compress the opposite nostril, and direct the patient to take long inhalations across the bottle's mouth. Do not tolerate spasmodic or jerky breathing. When an inhalation has occurred remove the bottle, so that nothing exhaled shall enter to contaminate the chloroform. At first the bottle should be distant enough for only the faintest odor to be detected. At no time near enough to irritate the fibrilla of nerves spread out upon the Schneiderian membrane, the throat and lungs. Do not give peripheral nerves a shock. The medulla oblongata lies closely contiguous,and will respond to the irritation all too readily. Rememb̶er that the nerves of the mouth, nose, throat and lungs in their ultimate distribution, if on a plane, cover a space of twelve to fourteen hundred feet all readily accessible to the narcotic laden air. Nerve impulse largely controls the sanguineous circulation. The blood readily absorbs the drug, and its globules roll over each other to the heart, to be sent out to brain, viscera and ganglia. Again, I say avoid shock—the first, more common cause of death from chloroform. Steal over the peripheral sentinels, so gradually, so warily that they shall not fire an alarm to Trigemina and Medulla. As the long regular breathing goes on the bottle can be neared till stronger vapor is taken. Presently the eyelids will begin to droop or "wink lazily;" the muscles somewhat relax, and an *obtundure*, if I may coin the word, creeps over the nerves. In such a state I extracted my tooth, and in such a state I have for others taken many teeth (too many I now fear.) In such a condition patients will submit to merciless cutting or drilling sensitive dentine. In this condition the drill or bur can be carried to the live pulp, and the pulp be amputated and afterwards the patient will say, "I knew what you were doing, but it did not hurt." In the case of children they will sometimes moan and cry out, but after restoration express no resentment, and all dread of subsequent operations is dispelled.

You will ask, in what way does your mode differ from the ordinary? I answer, in the avoidance of shock, by giving large volumes of air carrying and attenuating the narcotic, and stopping short of full anæsthesia. To show how different the line of procedure from the ordinary, I wish to quote from Vol. III, American System of Dentistry, page 109, under the head of "Preparation of the Patient" (for inhaling chloroform) the writer says, "Owing to the irritant effect upon delicate cutaneous surfaces of chloroform when contact is close and constant, the face of the patient, especially about the mouth and nose, should be anointed with some fatty substance as a protective shield."

I do not hesitate to say that an operator or surgeon, who ever under any circumstances should apply chloroform so closely and continuously to mouth and nose as to require an unctuous shield, is no more fit to give it or to teach others to administer it than is a child to play with matches in a powder house.

Since writing the above, the April No. of the Dental, Surgical Microcosm has come into my hands, wherein I find my views ably supported. Dr. J. R. Simms speaking of anæsthetics says, "A true anæsthetic should not produce any irritation of the epiglottis, bronchial tubes, or pulmonary membranes." Dr. Hays of Pennsylvania advertises in the same number an apparatus whereby he measures the per cent. of narcotic in air, and begins with one per cent of chloroform and increases gradually and "scientifically" (?) till narcotism is produced. It may be classed as "New," and I am inclined to think it "Useful."

Is the way I have described safe and efficient as an obtundent, and practical for daily operative use? I answer with all the emphasis at my command, *yes, yes.*

The Rev. Dr. Lawrence, of Chicago, says of it after an operation upon exceedingly sensitive teeth: "I have carefully watched the effects upon myself of the use of chloroform while undergoing dental operations. Of course, I know that the application was not such as to be a full one. I have been conscious most if not absolutely all of the time, and yet I have had no pain, and what was better no fear of pain, and consequently none of that frightful nervous strain which is really more trying in the present and the future relation of the patient.

"In all cases I have not detected the slightest ill effect upon any function after leaving the dentist's chair, on the contrary, I experienced a physical sense of comfort and relief.

"I was in my childhood prejudiced against all anæsthetics and especially chloroform. I tried the experiment because of my nervous sensibility, and with reluctance and close inspection of every symptom. My experience has in my case fully justified and commended the application."

Dr. Homer Thomas, of Chicago, permits me to quote him as giving hearty approval of such a use of chloroform. By him I was authorized to so employ it for his wife and children.

Dr. E. Fletcher Ingalls, of Chicago, Professor of diseases of the throat and chest in Rush Medical College, and in the Northwestern University Woman's Medical School, pays me an unusual and esteemed compliment by permitting the use of his name in endorsement in such a use of chloroform. He says:

"Having witnessed two deaths from chloroform I was naturally averse to its use in dental operations upon my family. However, yielding to the request of my wife that she might take it, and trusting to the care and experience of our dentist, I consented to the careful use of chloroform in this way, not only with her but with my children, and I am greatly pleased with the result. The operations which formerly caused the most intense suffering and dread are in this way easily borne and satisfactorily finished.

"I am greatly interested in this method of using this anæsthetic and have for years advised a similar plan which I first saw recommended by Balfour for the relief of the agony of Angina Pectoris. Thus used it appears perfectly safe, but I confess myself surprised to find it so effective in preventing pain."

During more than twelve past years, at my office, I have thus administered chloroform as an obtundent to all sorts of patients, (if I may thus describe them,) with no other ill effect than the slight nausea that I have described. I cannot truthfully claim immunity from ill results of operations where no obtundents have been permitted. Shock, and nervous illness have followed many times consequent upon operations when from prejudice I was not permitted its use, and that too, though I have long since learned the attendant danger and have gradually lessened the length of the sittings.

Two classes of dental patients call especially for avoidance of pain. Children from four years (even younger) to twelve or fourteen, and nervous persons, male and female, over that age. There are some persons of so stolid a make that they seem not to care for or fear any operation. They apparently have tendons for nerves, lymph for nerve fluid, adipose for periphera, and cuticular ganglia. Some such have astonished me and challenged my credulity by saying, "That the extraction of a tooth did not hurt." "It does not hurt to have my teeth filled." But such cases, as you all know, are rare. By far the greater number of patients cringe at the touch of an instrument to enamel, frighten before being hurt, moan and squirm at contact with sensitive dentine, weep and shiver, and grow hysterical, exhausting their own strength and that of the operator. It does not matter whether the suffering is imaginary or real. To the operator at least it is real. What operator is there who has not had his sympathies awakened by the child patient saying "Now Doctor will it hurt? Have you got to use the buzzer?" And from the older ones, who has not heard "Oh Doctor, I like you out of your office, but I hate to come for work, I hate your instuments. It just makes me sick." Who among you, gentlemen, have not, times without number, needed the poetized patience of Job while striving not to hurt, and accomplishing little. But I need not dwell upon this. It is a more than thrice told tale.

There is a view of this question that was recently presented to me in a forceful way. In a casual conversation with Rev. Dr. Lawrence, above quoted, it was claimed the dental profession was a long way in the rear of surgeons in the matter of the avoidance of pain in operations. The Doctor said: "Yes, it is true, you are not up to the level of surgery in pain avoidance. There is a brutishness of persistence in operating that in the public mind stamps the dental chair as a place of torture to be avoided until driven to it as a last dreaded resort." The charge is true. There was terse irony in it, a logic unanswerable.

What would be thought of a surgeon who in the light of today should perform laparotomy without an anæsthetic. You would all say he was a brute, and his operations, however skillful, brutish. We are amputating nerves, and performing operations that cause moans, entreaties, tears, shock often to syncope, sometimes collapse. Only week before last a young gentleman, on whom I was operating, fainted (having declined the help of chloroform.) After resuscitation was complete I said, "This will not do, if I operate for you, you must take chloroform, else I shall decline to serve you." "Is it safe?" "Yes, unsafe without it." "Then I'll trust you." From thence to finish the operation was painless, but consciousness was not for once suspended. On leaving the office he, with a pleased look, said "Doctor, next Monday I'll take the chloroform, it does not hurt." Whether he referred to the anæsthetic or the operation was not explained. In the case of children I absolutely refuse to operate painfully. Our profession does not demand baby torture.

Are there then no obstacles to the general adoption of the advocated practice? Yes, we have to sail under false colors, or meet a prejudice in the public mind whenever chloroform is named. It is no easy aversion to allay. It is founded upon recorded deaths from anæsthetics, all too numerous, in Chicago and New York, so recent as not to be forgotten, but I do not hesitate to say, needless deaths, caused by shock easily avoided, or by excessive anæsthetization. This prejudice, however, can be overcome by use of facts easily demonstrated. Clearly to my mind this should be done, or other means as good or better, sought out and adopted. There is surely in such a body of men as is before me, learning, skill and forcefulness sufficient to such an end. May the time soon come when the seekers for our skill and aid shall not be hindered by dread of "torture;" when to the gentle courtesy and dignified suavity, for which our profession is becoming noted, shall be added the charm of skilled and distinguished tenderness; when we can advertise painless operations, not by flaming posters and staring headlines, quackishly and unethically, but by quiet unpretentious putting aside prejudice by tender humanity, by acts which are benisons, by works which are so beneficent that we may clothe our offices with kindliness as ennobling to ourselves as it is inviting to our beneficiaries.

---

A NEW porcelain has been obtained by grinding asbestos to a fine powder and dissolving out all soluble matter with hydrochloric acid. The powder thus obtained is made into a paste with water and baked for eighteen hours at 1200 deg. in a furnace.

